# Dnmt3a-Mediated DNA Methylation Changes Regulate Osteogenic Differentiation of hMSCs Cultivated in the 3D Scaffolds under Oxidative Stress

**DOI:** 10.1155/2019/4824209

**Published:** 2019-11-15

**Authors:** Liangping Li, Zemin Ling, Wenwu Dong, Xiaoying Chen, Corina Vater, Hongxing Liao, Qihua Qi, Hao Hu, Yan Chen, Michael Gelinsky, Maik Stiehler, Xuenong Zou

**Affiliations:** ^1^Guangdong Provincial Key Laboratory of Orthopaedics and Traumatology, Orthopaedic Research Institute/Department of Spinal Surgery, The First Affiliated Hospital of Sun Yat-sen University, Guangzhou 510080, China; ^2^Centre for Translational Bone, Joint, and Soft Tissue Research, Medical Faculty and University Centre for Orthopaedics and Trauma Surgery, University Hospital Carl Gustav Carus at Technische Universität Dresden, Dresden 01307, Germany; ^3^Department of Orthopaedics, Sir Run Run Shaw Hospital, School of Medicine, Zhejiang University, Hangzhou 310016, China; ^4^Department of Orthopaedics, Shenzhen People's Hospital, 2nd Clinical Medical College of Jinan University, Shenzhen 518020, China; ^5^Department of Emergency, The Second Affiliated Hospital of Zhejiang University School of Medicine, Hangzhou 310009, China; ^6^Department of Orthopaedics, Meizhou People's Hospital, Meizhou 514000, China

## Abstract

Oxidative stress (OS) caused by multiple factors occurs after the implantation of bone repair materials. DNA methylation plays an important role in the regulation of osteogenic differentiation. Moreover, recent studies suggest that DNA methyltransferases (Dnmts) are involved in bone formation and resorption. However, the effect and mechanism of DNA methylation changes induced by OS on bone formation after implantation still remain unknown. Three-dimensional (3D) cell culture systems are much closer to the real situation than traditional monolayer cell culture systems in mimicking the *in vivo* microenvironment. We have developed porous 3D scaffolds composed of mineralized collagen type I, which mimics the composition of the extracellular matrix of human bone. Here, we first established a 3D culture model of human mesenchymal stem cells (hMSCs) seeded in the biomimetic scaffolds using 160 *μ*M H_2_O_2_ to simulate the microenvironment of osteogenesis after implantation. Our results showed that decreased methylation levels of ALP and RUNX2 were induced by H_2_O_2_ treatment in hMSCs cultivated in the 3D scaffolds. Furthermore, we found that Dnmt3a was significantly downregulated in a porcine anterior lumbar interbody fusion model and was confirmed to be reduced by H_2_O_2_ treatment using the 3D *in vitro* model. The hypomethylation of ALP and RUNX2 induced by H_2_O_2_ treatment was abolished by Dnmt3a overexpression. Moreover, our findings demonstrated that the Dnmt inhibitor 5-AZA can enhance osteogenic differentiation of hMSCs under OS, evidenced by the increased expression of ALP and RUNX2 accompanied by the decreased DNA methylation of ALP and RUNX2. Taken together, these results suggest that Dnmt3a-mediated DNA methylation changes regulate osteogenic differentiation and 5-AZA can enhance osteogenic differentiation via the hypomethylation of ALP and RUNX2 under OS. The biomimetic 3D scaffolds combined with 5-AZA and antioxidants may serve as a promising novel strategy to improve osteogenesis after implantation.

## 1. Introduction

Although bone repair materials have developed rapidly and are widely used in the clinic, the development of a strategy for improving osteogenesis remains a big challenge in the field of orthopaedics. Bone formation involves the recruitment, commitment, proliferation, and osteogenic differentiation of mesenchymal stem cells (MSCs) [1]. *In vivo* bone formation after implantation of bone repair materials is a more complex process that is influenced by oxidative stress (OS), inflammation response, and vascularization [2]. MSCs initially migrate around the bone repair materials and subsequently undergo hypoxia stress, OS, and even endoplasmic reticulum stress after implantation. Thereafter, the minority of MSCs fail to maintain homeostasis and finally become apoptotic or even necrotic because these stress reactions are too dramatic. However, the majority of MSCs are capable of bringing about a series of adaptive reactions that enable them to survive, proliferate, differentiate, and ultimately achieve osteogenesis due to an appropriate stress intensity.

OS, triggered by multiple factors, including ischemia, hypoxia, and inflammation, refers to the excessive accumulation of reactive oxygen species (ROS) that results from an imbalance between the generation and scavenging of ROS [3]. To defend themselves against OS, organisms possess inherent defence systems, including antioxidant enzymes and antioxidants [4]. Excessive ROS damage nucleic acids, proteins, and lipids and are associated with the pathology of numerous diseases [5], including bone nonunion [6] and osteoporosis [7]. Our previous *in vitro* experiments have demonstrated that titanium alloys, one of bone repair materials most frequently used in orthopaedics, can give rise to increased intracellular ROS production [8]. In addition, Tsaryk et al. [9] found that human endothelial cells seeded on a titanium alloy possess the ability to keep redox homeostasis to a certain extent. Furthermore, *in vivo* animal experiments by others [10–12] have shown that OS occurs most fiercely at the early stage and then gradually reaches redox homeostasis during fracture healing. However, the precise mechanisms by which bone formation occurs under OS after implantation are still elusive.

DNA methylation is crucial for a variety of physiological activities, including gene silencing, genomic imprinting, chromatin modification, and X chromosome inactivation [13]. DNA methylation predominantly occurs at CpG dinucleotides [14]. DNA methylation is mediated by several known DNA methyltransferases (Dnmts), including maintenance enzyme Dnmt1 and de novo methyltransferases Dnmt3a/3b [15]. Many diseases, such as autoimmune disorders and cancers, have been indicated to be associated with the aberration of genomic DNA methylation [16, 17]. Moreover, DNA methylation plays an important role in osteoblastic differentiation of MSCs [18, 19]. Especially, two recent studies suggest that Dnmt3a is involved in bone formation and resorption [20, 21]. Meanwhile, recent several studies also have revealed that the alterations of genomic DNA methylation and Dnmts are induced by OS [22–24]. However, the effect of DNA methylation changes induced by OS on osteogenic differentiation after implantation has been less studied.

Monolayer culture systems have played a key role in the field of bone physiology and in other fields of cellular biology. However, it is well known that cell morphology and activities, such as adhesion, migration, proliferation, and differentiation, in a flat two-dimensional (2D) condition are inconsistent with *in vivo* real situations. In contrast, three-dimensional (3D) cell culture systems are obviously superior to traditional monolayer cell culture systems in the simulation of the *in vivo* microenvironment, including the extracellular matrix, cell-cell interactions, and signal transduction [25, 26]. We have developed porous 3D scaffolds composed of mineralized collagen type I, a nanocomposite which mimics the composition of the extracellular matrix of the human bone [27]. The porous mineralized collagen 3D scaffolds fulfil a number of superior properties, including excellent biocompatibility, high interconnective porosity, and certain mechanical strength. The scaffolds have been confirmed to be suitable for the proliferation and osteogenic differentiation of MSCs by cell experiments [28] and confirmed to be suitable for bone formation by animal experiments [29]. Overall, the biomimetic 3D scaffolds combined with MSCs and drugs can be widely used for bone tissue engineering.

Our previous studies have demonstrated that homeostasis-related genes and epigenetic regulation, including miRNAs and DNA methylation, play an important role in the process of bone formation after implantation in the porcine anterior lumbar interbody fusion (ALIF) model [30–33]. In order to better understand the mechanisms by which *in vivo* bone formation occurs under OS after implantation, we first aimed to establish a 3D cell culture model mimicking redox homeostasis and osteogenesis after implantation. Furthermore, we investigated the effect and underlying mechanism of DNA methylation changes on the osteogenic differentiation of MSCs under OS using the 3D *in vitro* model. Moreover, we examined whether the Dnmt inhibitor could enhance the osteogenic differentiation of MSCs cultivated in the 3D scaffolds under OS, providing a theoretical basis for bone tissue engineering.

## 2. Materials and Methods

### 2.1. Chemicals and Reagents

All chemicals (H_2_O_2_, 3-(4,5-dimethylthiazol-2-yl)-2,5-diphenyltetrazolium bromide (MTT), dexamethasone, *β*-glycerophosphate, ascorbic acid, p-nitrophenylphosphate (pNPP), 2′,7′-dichlorofluorescein diacetate (DCFH-DA), 4% paraformaldehyde, Triton X-100, and 5′-AZA-2′-deoxycytidine (5-AZA)) were purchased from Sigma-Aldrich (Taufkirchen, Germany). 4′,6-Diamidino-2-phenylindole, dihydrochloride (DAPI) and Goat anti-mouse Alexa Fluor® 546 were purchased from Invitrogen/Thermo Fisher Scientific (Carlsbad, CA, USA). The primary antibody anti-Dnmt3a monoclonal mouse lgG was purchased from R&D Systems (Minneapolis, MN, USA).

### 2.2. Cell Seeding and Cell Culture in the Porous 3D Scaffolds

hTERT-transduced human bone marrow-derived MSCs (hMSCs) were purchased from Cambrex Corporation (East Rutherford, NJ, USA) and characterized as shown before [34]. Cells were cultivated in Dulbecco's Modified Eagle's Medium low glucose (Biochrom, Berlin, Germany), containing 10% fetal bovine serum (Biochrom, Berlin, Germany), 10 U/mL penicillin, and 100 *μ*g/mL streptomycin (Biochrom, Berlin, Germany) in an incubator (37°C, 5% CO_2_, and 95% humidity).

Cylindrical porous scaffolds made from mineralized collagen with a diameter of 6 mm and a height of 3 mm were used in the present study. The scaffolds were preincubated with cell culture medium for 24 h before cell seeding. Thereafter, equilibration medium was removed and the scaffolds were placed on sterile filter paper to remove excess liquid from the pores. For cell seeding, the method was optimized on the basis of our prior study [35]. The scaffolds were placed in 24-well polystyrene culture dishes, and 50 *μ*L cell suspensions with 2 × 10^5^ cells were pipetted onto the top of each scaffold. After 30 min of initial adhesion, 500 *μ*L of the cell culture medium was carefully added to 24-well culture dishes per scaffold. After 24 h, cell-seeded scaffolds were transferred to fresh culture dishes.

### 2.3. MTT Staining

After the cultivation of the cell-seeded scaffolds for 24 h, the culture medium was removed. The samples were rinsed with PBS and then placed on sterile filter paper to remove excess liquid. Thereafter, the samples were incubated with 1.2 mM MTT for 4 h at 37°C. The formation of dark blue formazan dye converted from MTT by mitochondrial dehydrogenases of living cells was documented photographically.

### 2.4. Cell Treatment

Different concentrations of hydrogen peroxide (H_2_O_2_) were added to induce OS after hMSCs were seeded in the 3D scaffolds and cultivated for 24 h. For osteogenic differentiation, fresh osteogenic medium (OM; 0.1 *μ*M dexamethasone, 50 *μ*g/mL ascorbic acid, and 10 mM *β*-glycerophosphate) was used for osteogenic induction after the cells reached 70–80% confluence. The medium was changed every three days. After removing the medium and washing twice with warm PBS, cell-seeded scaffolds were placed on filter paper to remove excess liquid and then frozen at -80°C until further analysis (see latter section).

### 2.5. Cellular Viability

Cell viability was probed using a lactate dehydrogenase (LDH) assay. LDH is a stable cytosolic enzyme present in all cell types. The cellular viability can be measured in terms of LDH released from dead cells into the supernatant upon the rupture of the cell membrane. For this purpose, the CytoTox 96® Nonradioactive Cytotoxicity Assay Kit (Promega, Madison, WI, USA) was employed according to the manufacturer's instructions. The cell-seeded scaffolds were transferred to a new 48-well plate and 500 *μ*L 1% Triton X-100 was added to each well to induce maximum LDH release. The scaffolds were smashed after cell lysis for 30 min at room temperature (RT). After centrifugation, an aliquot of 200 *μ*L supernatant per well was transferred to a new corresponding tube. To define the LDH content, 50 *μ*L of supernatant with 50 *μ*L of substrate solution was mixed in 96-well plates. After 30 min of incubation at RT in the dark, the enzymatic reaction was stopped with 50 *μ*L of stop solution. The absorbance was measured spectrophotometrically at 490 nm using a microplate reader (SpectraFluor Plus, Tecan).

### 2.6. ROS Assessment

Intracellular ROS was measured using a method previously described with a minor modification [36]. In brief, after the hMSCs were exposed to different concentrations of H_2_O_2_ for 1 h or 24 h, the cell-seeded scaffolds were transferred to a new 48-well plate. The samples were incubated with 500 *μ*L medium containing 20 *μ*M DCFH-DA in each well for 30 min at 37°C. After the removal of the medium containing DCFH-DA, the cells were rinsed with PBS three times to remove the residual extracellular DCFH-DA. After 500 *μ*L PBS was added per well, the scaffolds were smashed and mixed. An aliquot of 100 *μ*L supernatant per well was transferred to a new 96-well opaque black plate after centrifugation. The fluorescence levels were determined using a fluorescence microplate reader with the excitation and emission wavelengths set at 488 and 525 nm, respectively.

### 2.7. Determination of DNA Content and ALP Activity

The medium was removed and rinsed twice with PBS after osteogenic induction for 14 days. The scaffold was transferred to a new 48-well plate and stored at -80°C until measurement. To evaluate the ALP activity, 500 *μ*L 1% Triton X-100 was added in each well to fully lyse cells for 50 min at RT after the frozen samples were thawed on ice for 20 min. Thereafter, the scaffolds were smashed and centrifugation was performed. An aliquot of 25 *μ*L cell lysates was added to an ALP reaction buffer (1 mg/mL pNPP, 0.1 M diethanolamine, 1% Triton X-100, and 1 mM MgCl_2_; pH 9.8) and incubated for 30 min at 37°C. The enzymatic reaction was quenched by adding 63 *μ*L 1 N NaOH. The absorbance was measured at 405 nm with a multifunction microplate reader (SpectraFluor Plus, Tecan). A calibration line was made according to different dilutions of 1 mM p-nitrophenol stock solution. For DNA assay, one aliquot of 10 *μ*L cell lysates was used to determine the DNA content using the PicoGreen dsDNA quantification reagent (Molecular Probes) according to the manufacturer's instructions. The intensity of fluorescence was measured with the multifunction microplate reader mentioned above at an excitation and emission wavelength of 485/535 nm. DNA content was correlated with relative fluorescence units using a calibration line. The ALP activity was expressed as nmol/min/*μ*g DNA.

### 2.8. Cell Immunofluorescence

#### 2.8.1. 2D Coverslip Condition

Approximately 5000 hMSCs were plated onto cover slips. The cells were incubated with 160 *μ*M H_2_O_2_ for 24 h after being cultivated for 24 h. Cells were fixed with 4% paraformaldehyde solution for 20 min and then rinsed with PBS. For permeabilization, cells were incubated with 1% Triton X-100 PBS solution for 20 min at RT. After washing with PBS, unspecific binding sites were blocked with blocking buffer (5% goat serum in PBS) for 45 min at RT. Then, cells were incubated with primary antibody anti-Dnmt3a (10 *μ*g/mL) overnight at 4°C. After washing three times with PBS, cells were incubated with secondary antibody solution (Alexa Fluor® 546 goat anti-mouse) diluted 1 : 500 in PBS solution for 1 h in the dark at RT. Nuclei were counterstained by incubation with DAPI (1 *μ*g/mL) for 5 min at RT. After washing three times to remove unbound DAPI, the stained cells were mounted with mounting medium. Images of the staining were taken with a fluorescence microscope (VB-6000, Keyence, Japan) under standardized conditions and analyzed with ImageJ 1.45s software.

#### 2.8.2. 3D Scaffold Condition

Briefly, 2.5 × 10^5^ hMSCs were seeded in each scaffold. The scaffolds were cut into two halves from the middle sagittal plane using a scalpel. 50 *μ*L of anti-Dnmt3a (25 *μ*g/mL) was added from the cutting face of each scaffold and incubated overnight at 4°C. Images of the staining were taken using a Leica scanning confocal microscope (Leica Microsystems, Wetzlar, Germany). The remaining steps are the same as described above in 2D Coverslip Condition.

### 2.9. Cell Transfection

A Dnmt3a overexpression plasmid, pcDNA3.1-Dnmt3a, was commercially constructed by Hanbio (Shanghai, China), and an empty pcDNA 3.1 vector (NC) was used as the control. 2.5 × 10^5^ hMSCs were seeded in each scaffold and transfected with 2 *μ*g pcDNA3.1-Dnmt3a plasmid or control vector pcDNA3.1-NC in Opti-MEM™ I Reduced Serum Medium using Lipofectamine™ 3000 (Invitrogen) according to the manufacturer's instructions. The medium was replaced by corresponding medium after transfection for 6 h.

### 2.10. Bisulfite Sequencing

Total genomic DNA was isolated using a Wizard® Genomic DNA Purification Kit (Promega, Madison, WI, USA) according to the manufacturer's instructions. Bisulfite conversion of isolated genomic DNA was carried out using a commercially available EpiTect™ Bisulfite Kit (Qiagen, Hilden, Germany) according to the manufacturer's protocol. To identify the methylation patterns, primers were designed to amplify the promoter region of ALP and RUNX2. PCR was performed using the Takara EpiTaq HS (Takara, Japan). The PCR products were purified using a DNA gel recovery kit (Dongsheng Biotech Co., Ltd., Guangzhou, China) and then cloned into the T-Vector pMD19-T. The recombinant plasmid was subsequently transformed into the *Escherichia coli* strain DH5*α*. Eight clones from each sample were sequenced by the GENEWIZ company (Suzhou, China). The number of total CpG sites in each sample was used as the denominator to evaluate the percentage alteration in the methylation of ALP and RUNX2.

### 2.11. RNA Isolation, cDNA Synthesis, and Real-Time PCR

Samples were rinsed with PBS, and excess PBS was removed with a filter paper. All samples were frozen immediately at -80°C until later use. Total RNA was isolated using the RNeasy Mini Kit (Qiagen, Hilden, Germany) according to the manufacturer's instructions. RNA concentration and quality were measured at 260 nm and 260/280 nm using a NanoDrop 2000c Spectrophotometer (Peqlab, Erlangen, Germany). cDNA synthesis from 200 ng RNA for each sample was performed using Superscript II Reverse Transcriptase and with oligo-dT (Invitrogen, Karlsruhe, Germany) according to the manufacturer's instructions. 1 *μ*L of total cDNA was amplified in a 10 *μ*L reaction mix containing 5 *μ*L of SYBR green (Life Sciences). Real-time PCR reactions were performed using a 7500 Fast Real-Time PCR System (Applied Biosystems). The expression levels of each mRNA were normalized to that of GAPDH, and the relative expression levels were calculated using the 2-ΔΔCt method. The primers used for amplification of the target mRNA are listed in Table 1.

### 2.12. Western Blot

Cells were harvested for protein extraction using RIPA buffer supplemented with PMSF. The protein was separated by SDS-PAGE and then transferred onto a 0.45 *μ*m PVDF membrane. The membranes were blocked and then incubated overnight with primary polyclonal anti-Dnmt3a (1 : 1000, Abcam) or GAPDH (1 : 3000, Abcam) antibodies. The antibodies were detected using enhanced chemiluminescence with HRP-conjugated secondary antibodies (Jackson ImmunoResearch Inc.). The values of the band intensities were quantified using ImageJ software.

### 2.13. Animal Model and Microarray Experiment

Detailed descriptions of the ALIF model and mRNA microarray experiment were elucidated in our previous study [30]. Briefly, eighteen Danish female landrace pigs, 3 months old, weighing approximately 50 kg, were used. A 3-level ALIF procedure was performed on each pig under general anaesthesia. The bone repair materials such as a polyetheretherketone interbody cage with equine bone protein extracts (COLLOSS® E; Ossacur AG, Oberstenfeld, Germany), rhBMP-2 dissolved on ACS (INFUSE®; Medtronic-Sofamor Danek, Memphis, TN), or autograft bone were randomly inserted into each operation segment. Each of the six pigs was killed at 2, 4, or 8 weeks using an overdose injection of pentobarbital (20 mg/kg). All animal operations and experiments complied with the Danish Law on Animal Experimentation and were approved by the Danish Ministry of Justice Ethical Committee, J.nr. 2004-561-898. The animal experiment was performed at the Institute of Clinical Medicine at Aarhus University. Gene expression profiling was carried out for each pooling RNA sample separately on the GeneChip® Porcine Genome Array (Affymetrix) at the CapitalBio Corporation (Beijing, China). The protocol for the microarray processing was performed according to the GeneChip Expression Analysis Technical Manual (Affymetrix, Rev. 5, Part number 701021).

### 2.14. Statistical Analysis

The data are presented as the mean ± SD. The difference between two groups was evaluated using Student's *t*-test. The difference among multiple groups was probed using a one-way ANOVA followed by Bonferroni's posttests. *P* values < 0.05 were considered to be statistically significant. Each experiment was repeated independently at least three times.

## 3. Results

### 3.1. Establishment of a 3D Cell Culture Model under OS

**Figure 1:**
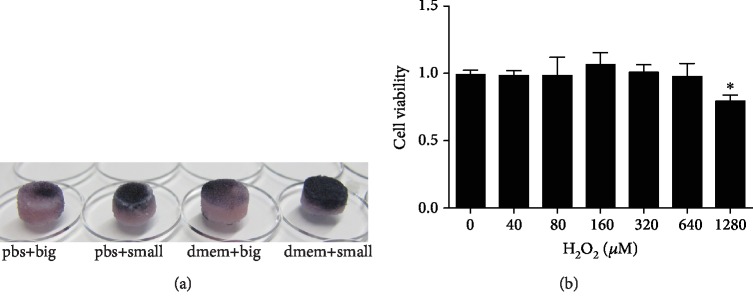
Establishment of a 3D cell culture model under OS. (a) The effect of different methods of cell seeding on the cell distribution of hMSCs in the 3D scaffolds. According to the types of cultivation methods, it was divided into four groups: pbs+big, pbs+small, dmem+big, and dmem+small. Namely, “big” and “small” refer to 500 *μ*L and 50 *μ*L cell suspensions with equal cell numbers (2.5 × 10^5^) added to the 3D scaffolds, respectively. After cells were seeded in the 3D scaffolds and cultured for 24 h, MTT staining of the whole scaffolds was performed. (b) Cell viability was determined using an LDH assay after hMSCs were incubated with 40, 80, 160, 320, 640, and 1280 *μ*M H_2_O_2_ for 24 h. 0 *μ*M H_2_O_2_ was used as the control group. ^∗^*P* < 0.05 compared to the control group. The results indicate the mean ± SD of triplicate experiments.

To establish a 3D culture model of hMSCs in the porous mineralized collagen scaffolds, the effect of different methods of cell seeding on the cell distribution in the 3D scaffolds was evaluated using MTT staining. According to the types of cultivation methods, it was divided into four groups: pbs+big, pbs+small, dmem+big, and dmem+small. After hMSCs were seeded in the 3D scaffolds and cultured for 24 h, MTT staining was performed. MTT staining indicated that the seeding method “small” was significantly better than the seeding method “big.” After only 24 h of cultivation, hMSCs penetrated from the surface through less than 25% depth of the scaffolds in the “dmem+big” group, whereas hMSCs penetrated from the surface through more than a 50% depth of the scaffolds in the “dmem+small” group (Figure 1(a)). Therefore, we employed the cell seeding method “dmem+small” to establish a 3D culture model of hMSCs in the biomimetic scaffolds for mimicking an *in vivo* cell growth environment.

Next, cell viability was examined using an LDH assay. hMSCs were treated with different concentrations of H_2_O_2_ for 24 h. Cell viability did not significantly decrease when hMSCs were incubated with H_2_O_2_ at concentrations of no more than 1280 *μ*M (Figure 1(b)).

### 3.2. Establishment of the 3D Model Mimicking Redox Homeostasis and Osteogenesis after Implantation

**Figure 2:**
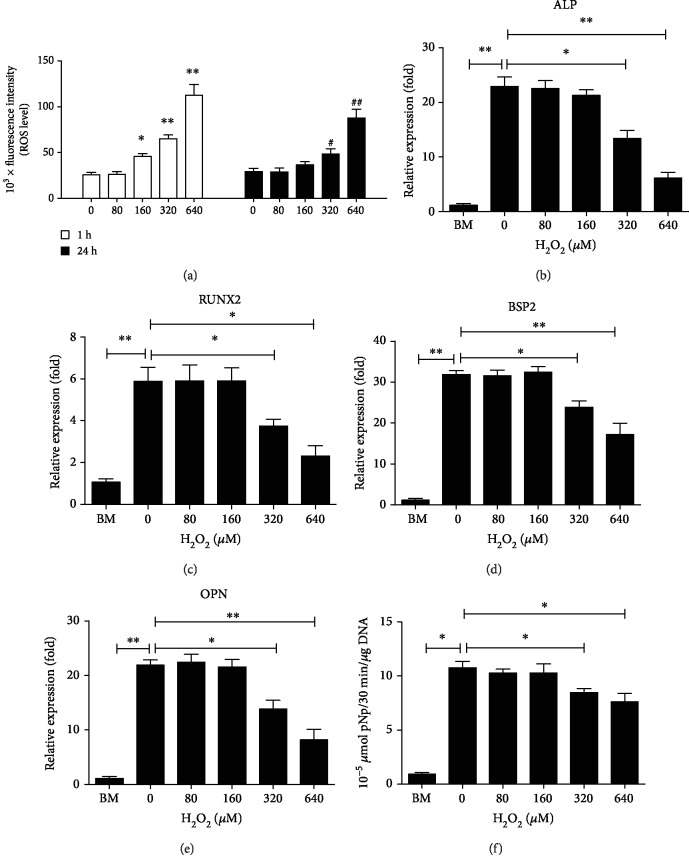
The levels of intracellular ROS and osteogenic differentiation of hMSCs seeded in the 3D scaffolds under OS. (a) The levels of intracellular ROS at 1 h and 24 h were determined after hMSCs were exposed to 80, 160, 320, and 640 *μ*M H_2_O_2_. The fluorescence intensity of ROS was determined using a fluorescence microplate reader. 0 *μ*M H_2_O_2_ at 1 h and 24 h were used as the control group. ^∗^*P* < 0.05 and ^∗∗^*P* < 0.01 compared to the control group at 1 h. ^#^*P* < 0.05 and ^##^*P* < 0.01 compared to the control group at 24 h. (b, c, d, and e) The cells were incubated with basal medium (BM) or osteogenic medium (OM) containing 80, 160, 320, and 640 *μ*M H_2_O_2_ for 7 days after hMSCs were seeded in the 3D scaffolds. Thereafter, qRT-PCR was performed to evaluate the expression of ALP, RUNX2, BSP, and OPN, several key osteogenic markers. (F) ALP activity was measured after hMSCs were incubated with BM or OM containing 80, 160, 320, and 640 *μ*M H_2_O_2_ for 14 days in the 3D scaffolds. ^∗^*P* < 0.05 and ^∗∗^*P* < 0.01. The results indicate the mean ± SD of triplicate experiments.

To mimic redox homeostasis and osteogenesis after the implantation of bone repair materials, intracellular ROS at different times were first determined. 40 and 1280 *μ*M H_2_O_2_ were excluded because of the too low and too high concentrations, respectively. H_2_O_2_ treatment resulted in increased levels of intracellular ROS at both 1 h and 24 h in a dose-dependent manner in hMSCs seeded in the 3D scaffolds. Compared with the control group, the levels of intracellular ROS at 1 h significantly increased when hMSCs were treated with 160 *μ*M H_2_O_2_. However, no significant increase was observed at 24 h (Figure 2(a)).

In addition, we investigated the effect of H_2_O_2_ treatment on osteogenic differentiation of hMSCs seeded in the 3D scaffolds. After hMSCs were exposed to 160 *μ*M H_2_O_2_ and then incubated with osteogenic medium (OM), we employed real-time quantitative polymerase chain reaction (qRT-PCR) to detect osteogenic marker genes. Our findings showed that the mRNA expression of ALP, RUNX2, BSP2, and OPN, several key osteogenic markers, significantly decreased in a dose-dependent manner when hMSCs were exposed to H_2_O_2_ at concentrations of more than 160 *μ*M. However, they did not decrease for H_2_O_2_ at concentrations of no more than 160 *μ*M (Figures 2(b)–2(e)). To further confirm these findings, alkaline phosphatase (ALP) activity was assessed. We found that ALP activity changed in a manner consistent with ALP mRNA expression (Figure 2(f)). Taken together, these results suggested that the levels of intracellular ROS increased at an early stage, then gradually decreased to normal levels, and finally differentiated towards osteoblasts when hMSCs were incubated with 160 *μ*M H_2_O_2_ and OM. Thus, hMSCs exposed to 160 *μ*M H_2_O_2_ and then incubated with OM in the biomimetic scaffolds were used as a 3D cell model to simulate redox homeostasis and osteogenesis after implantation in the following study.

### 3.3. Decreased Methylation of ALP and RUNX2 under OS

**Figure 3:**
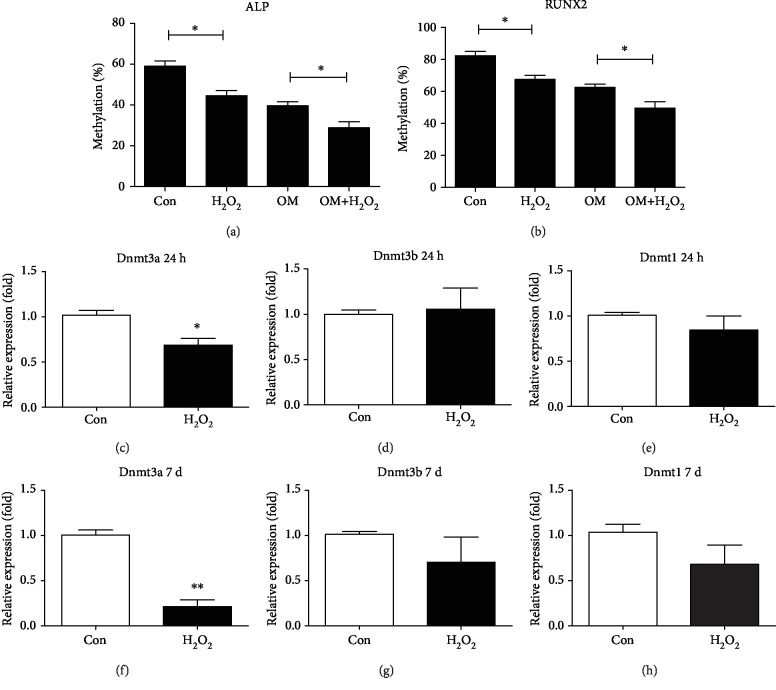
Decreased methylation of ALP and RUNX2 and the mRNA expression of Dnmts under OS. (a, b) The methylation percentages of the ALP and RUNX2 promoter region were measured using bisulfite sequencing after hMSCs were incubated with basal medium (BM) or osteogenic medium (OM) containing 160 *μ*M H_2_O_2_ for 7 days in the 3D scaffolds. (c, d, e, f, g, and h) The mRNA expression levels of Dnmt3a, Dnmt3b, and Dnmt1 at 24 h and 7 days were measured using qRT-PCR after hMSCs were continuously exposed to 160 *μ*M H_2_O_2_ in the 3D scaffolds. 0 *μ*M H_2_O_2_ was used as the control group. ^∗^*P* < 0.05 and ^∗∗^*P* < 0.01. The results indicate the mean ± SD of triplicate experiments.

To examine whether the upregulations of ALP and RUNX2 expression are accompanied by the reductions in DNA methylation levels during osteogenic differentiation of hMSCs under OS, the DNA methylation status of ALP and RUNX2 promoter region was determined using bisulfite sequencing after hMSCs were incubated with basal medium (BM) or OM containing H_2_O_2_. Compared with nonosteogenic differentiated (non-OD) hMSCs, the methylation levels of both ALP and RUNX2 were significantly lower in osteogenic differentiated (OD) hMSCs. Moreover, the methylation levels of both ALP and RUNX2 were significantly decreased by H_2_O_2_ treatment. In detail, the methylation levels of the ALP promoter region were reduced from 59.0% to 44.6% for non-OD hMSCs and from 39.5% to 28.9% for OD hMSCs, respectively (Figure 3(a)). The methylation levels of the RUNX2 promoter region were reduced from 82.4% to 67.4% for non-OD hMSCs and from 62.9% to 49.7% for OD hMSCs, respectively (Figure 3(b).

### 3.4. Downregulation of Dnmt3a Expression Induced by OS

Our previous study indicated that genes and epigenetics, including microRNAs, play an important role in osteogenesis after implantation using an ALIF model of pigs [30–32]. To explore the reason why the methylation levels of ALP and RUNX2 were decreased under OS, we examine the expression of Dnmts, including Dnmt3a, Dnmt3b, and Dnmt1, in the porcine ALIF model using microarray technology. We found that Dnmt3a expression at 2 weeks was significantly lower compared to that at 4 and 8 weeks, and the expression of Dnmt3b and Dnmt1 was not altered in the ALIF model (Table 2), suggesting that Dnmt3a expression decreased at an early stage after implantation when ROS were at their peak.

To confirm the expression of Dnmts under OS, qRT-PCR was performed after hMSCs were incubated with 160 *μ*M H_2_O_2_ for 24 h and 7 days. Compared with the control group, the mRNA levels of Dnmt3a in the H_2_O_2_ group at 24 h were downregulated (Figure 3(c)), whereas, the mRNA levels of Dnmt3b and Dnmt1 in the H_2_O_2_ group at 24 h did not significantly decrease (Figures 3(d) and 3(e)). In addition, H_2_O_2_ treatment resulted in lower mRNA levels of Dnmt3a at 7 days (Figure 3(f)). Although H_2_O_2_ treatment resulted in a slight decrease in the mRNA levels of Dnmt3b and Dnmt1 at 7 days, the difference was not statistically significant (Figures 3(g) and 3(h)).

**Figure 4:**
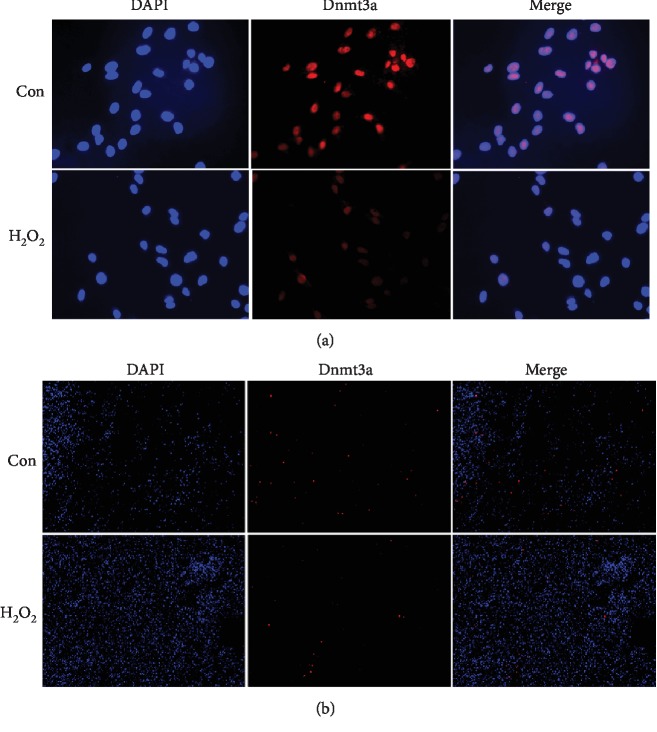
Downregulation of Dnmt3a protein expression under OS. The protein levels of Dnmt3a were measured using cell immunofluorescence after hMSCs seeded in the 24-well plate (a) or 3D scaffolds (b) were incubated with 160 *μ*M H_2_O_2_ for 24 h. 0 *μ*M H_2_O_2_ was used as the control group. The images shown are representative of three independent experiments.

**Figure 5:**
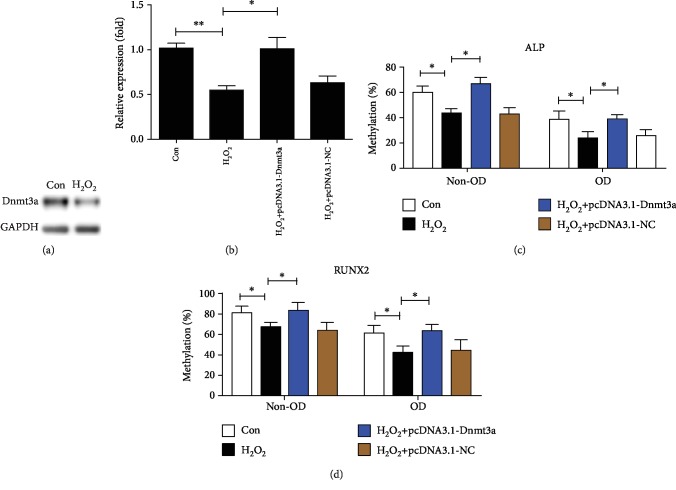
Dnmt3a protein expression and Dnmt3a rescue under OS. (a) The protein levels of Dnmt3a were measured using Western blot after hMSCs seeded in the 3D scaffolds were incubated with 160 *μ*M H_2_O_2_ for 24 h. Recombinant plasmid pcDNA3.1-Dnmt3a was commercially constructed and transferred into hMSCs using Liposome 3000. (b) qRT-PCR shows the mRNA levels of Dnmt3a at 3 days under OS after transfection of pcDNA3.1-Dnmt3a or pcDNA3.1-NC. (c, d) hMSCs seeded in the 3D scaffolds were incubated with basal medium (BM) or osteogenic medium (OM) containing 160 *μ*M H_2_O_2_ for 3 days after transfection. The methylation percentages of the ALP and RUNX2 promoter region were measured using bisulfite sequencing. 0 *μ*M H_2_O_2_ was used as the control group, and pcDNA3.1-NC was used as negative control. ^∗^*P* < 0.05 and ^∗∗^*P* < 0.01. The results indicate the mean ± SD of triplicate experiments.

Furthermore, we examined the protein expression of Dnmt3a under OS using cell immunofluorescence. Because it is more difficult to perform cell immunofluorescence for hMSCs seeded in the 3D scaffolds, we first carried it out using the 24-well plate. Compared with the control group, decreased protein expression of Dnmt3a in the H_2_O_2_ group was observed when hMSCs were seeded on the 24-well plate (Figure 4(a)). Similarly, the protein expression of Dnmt3a was downregulated in response to H_2_O_2_ treatment when hMSCs were seeded in the 3D scaffolds (Figure 4(b)). Moreover, H_2_O_2_ treatment resulted in reduced protein levels of Dnmt3a when hMSCs were seeded in the 3D scaffolds (Figure 5(a)). Collectively, these findings indicate that Dnmt3a, but not Dnmt3b or Dnmt1, was downregulated by OS and may mediate the hypomethylation of ALP and RUNX2.

### 3.5. Hypomethylation of ALP and RUNX2 Were Abolished by Dnmt3a Overexpression under OS

To investigate whether Dnmt3a mediated the hypomethylation of ALP and RUNX2 under OS, recombinant plasmid pcDNA3.1-Dnmt3a was constructed and transferred into hMSCs using Liposome 3000. Transfection of pcDNA3.1-Dnmt3a led to increased mRNA levels of Dnmt3a under OS as shown by qRT-PCR (Figure 5(b)). Decreased methylation levels of ALP and RUNX2 induced by H_2_O_2_ treatment were abolished by Dnmt3a overexpression. The methylation levels of the ALP promoter region were increased from 42.9.0% to 66.2% for non-OD hMSCs and from 23.5% to 38.5% for OD hMSCs, respectively (Figure 5(c)). The methylation levels of the RUNX2 promoter region were upregulated from 66.8% to 85.8% for non-OD hMSCs and from 43.0% to 67.4% for OD hMSCs, respectively (Figure 5(d)). Taken together, these findings indicate that Dnmt3a may mediate the hypomethylation of ALP and RUNX2 under OS.

### 3.6. AZA Enhanced Osteogenic Differentiation by Hypomethylation of ALP and RUNX2 under OS

**Figure 6:**
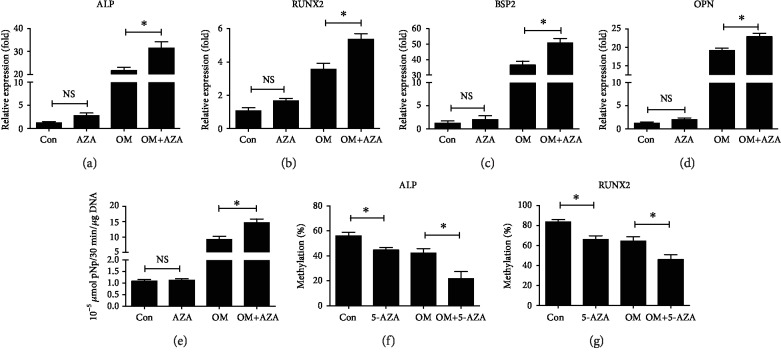
AZA enhanced osteogenic differentiation by the hypomethylation of ALP and RUNX2 under OS. hMSCs were pretreated with 10 *μ*M 5-AZA for 24 h after seeding in the 3D scaffolds. Afterwards, the cells were incubated with basal medium (BM) or osteogenic medium (OM) containing 160 *μ*M H_2_O_2_ for the indicated times. (a, b, c, d) The mRNA expression of ALP, RUNX2, BSP, and OPN at 7 days was evaluated using qRT-PCR. (e) ALP activity was determined at 14 days. (f, g) The methylation percentages of the ALP and RUNX2 promoter region at 7 days were measured using bisulfite sequencing. 0 *μ*M H_2_O_2_ was used as the control group. ^∗^*P* < 0.05. ns: not significant. The results indicate the mean ± SD of triplicate experiments.

5-AZA is a typical Dnmt inhibitor that is widely used to reduce the levels of DNA methylation [37]. Previous studies reported by others suggest that 5-AZA can enhance osteogenic differentiation in a 2D culture condition [38, 39]. Therefore, we examined whether 5-AZA can enhance osteogenic differentiation of hMSCs seeded in the 3D scaffolds under OS. 5-AZA leads to a slight increase of ALP and RUNX2 mRNA expression after hMSCs were exposed to H_2_O_2_ without OM; however, the difference is not significant (Figures 6(a) and 6(b)). The mRNA expressions of ALP, RUNX2, BSP, and OPN were significantly increased in response to 5-AZA treatment in OD hMSCs exposed to H_2_O_2_ (Figures 6(a)–6(d)). Accordingly, ALP activity was raised by 5-AZA in a manner consistent with the levels of ALP mRNA (Figure 6(e). Furthermore, the methylation levels of ALP and RUNX2 were determined after hMSCs were treated with 5-AZA and then incubated with H_2_O_2_ and OM. 5-AZA led to decreased ALP methylation levels from 56.0% to 44.7% for non-OD hMSCs and from 42.3% to 21.9% for OD hMSCs, respectively (Figure 6(f)). 5-AZA led to a decrease in the levels of RUNX2 methylation from 83.6% to 66.0% for non-OD hMSCs and from 64.6% to 46.0% for OD hMSCs, respectively (Figure 6(g)). Taken together, these results suggest that 5-AZA can enhance osteogenic differentiation of hMSCs seeded in the 3D scaffolds under OS probably through the hypomethylation of ALP and RUNX2 that is correlated with an increased expression of ALP and RUNX2.

## 4. Discussion

Although the molecular and cellular events required for *in vivo* bone formation after the implantation of bone repair materials are generally understood, the effect of DNA methylation changes on osteogenesis under OS is surprisingly poorly understood. To our best knowledge, the present study serves as the first study to document the effect of Dnmt3a-mediated DNA methylation changes on osteogenic differentiation under OS via establishing a novel 3D cell culture model using 160 *μ*M H_2_O_2_ to mimic the microenvironment after implantation. Furthermore, we found that the hypomethylation of ALP and RUNX2 under OS may be mediated by Dnmt3a, but not Dnmt3b or Dnmt1. Moreover, we confirmed that the Dnmt inhibitor 5-AZA can enhance osteogenic differentiation of hMSCs probably via the hypomethylation of ALP and RUNX2 in the context of OS. Due to the complexity of *in vivo* bone formation and the difficulty of 3D cell culture in the scaffolds, there are two important considerations with regard to these observations. First, it is difficult to establish a cell culture model to completely simulate the microenvironment of bone formation after implantation. Second, it is also difficult to elaborately investigate the profound mechanism using a 3D cell culture model. Despite these considerations, the present 3D cell culture model can be used to roughly explore the effect and underlying mechanism of DNA methylation changes on bone formation under OS after implantation.

Over the past decades, osteogenesis has been extensively studied using the traditional 2D culture systems. Although these traditional cultures can be still useful to investigate the molecular mechanisms of the regulation of osteogenesis, it is currently obvious that this approach cannot be utilized to convincingly mimic the process of bone formation. In particular, the complex organization between cells and the extracellular matrix strictly relies on the 3D surroundings [40]. To better stimulate the microenvironment of *in vivo* bone formation after implantation, a 3D cell culture model using the biomimetic scaffolds was established in the study. The 3D scaffolds with an interconnecting porosity of 72% and a mean pore diameter of 180 *μ*m have been shown to be highly suitable for homogenous MSC seeding. We found that hMSCs can penetrate from the surface through much deeper scaffolds after 14 days using the same seeding method and cell number as “small” in our previous study [35]. To facilitate cell penetration throughout the scaffolds, we optimized the scaffolds. Both the height and diameter of the scaffolds were reduced by about half in the present study. It was shown that there is active penetration of hMSCs from the surface through more than a 50% depth of the scaffolds after only 24 h using the seeding method “small” in the present study. It is reasonable to deduce that hMSCs can penetrate from the surface throughout the whole scaffolds after 14 days in the present study. Therefore, we considered that the seeding method “small” is a simple and effective way to establish a 3D culture model of hMSCs in the biomimetic scaffolds for mimicking the *in vivo* surroundings of osteogenesis.

Previous animal experiments by others have demonstrated that OS occurs fiercely at an early stage and then redox homeostasis is gradually achieved during fracture healing. In detail, the marker of OS increased at days 7 and 14 and then returned to basal levels at day 28 during fracture healing in a rat model [10]. Another study showed that the OS marker increased temporarily for 4 weeks and that an 8-week period was sufficient to reestablish redox homeostasis during bone formation after implantation in a rabbit tibia model [11]. To simulate *in vivo* redox homeostasis after implantation, we found that 160 *μ*M H_2_O_2_ treatment for 1 h resulted in a significant increase in the levels of intracellular ROS; however, there was no significant increase after 24 h in the present study. Moreover, our present study elaborately demonstrated that treatment with 160 *μ*M H_2_O_2_ did not inhibit osteogenic differentiation of hMSCs, as evidenced by unchanged osteogenic marker genes and ALP activity. However, treatment with 320 and 640 *μ*M H_2_O_2_ significantly inhibits osteogenic differentiation, as shown by decreased osteogenic marker genes and ALP activity. Taken together, our results suggested that hMSCs exposed to 160 *μ*M H_2_O_2_ initially underwent OS, subsequently reached redox homeostasis, and finally achieved osteogenic differentiation. Thus, it is reasonable to consider that the 3D cell culture model using 160 *μ*M H_2_O_2_ and OM can mimic the scenario of redox *in vivo* homeostasis and osteogenesis after implantation.

Recent studies have also demonstrated that OS regulated genomic DNA methylation, as well as Dnmts [22, 23]. Consistent with these studies, we found that the methylation levels of the ALP and RUNX2 promoter regions were reduced by H_2_O_2_ treatment in both undifferentiated hMSCs and differentiated hMSCs using bisulfite sequencing. Furthermore, we found that Dnmt3a expression at 2 weeks was lower than that at 4 weeks and 8 weeks in the porcine ALIF model using microarray technology, whereas the levels of Dnmt3b and Dnmt1 expression at 2 weeks were not different from that at 4 weeks or 8 weeks. In addition, we confirmed that only Dnmt3a mRNA, but not Dnmt3b or Dnmt1 mRNA, was downregulated by H_2_O_2_ treatment for 24 h and 7 days. Dnmt3a protein expression was verified to be reduced in response to H_2_O_2_ treatment both in a flat 2D condition and 3D condition using cell immunofluorescence and Western blot. What's more, the hypomethylation of ALP and RUNX2 induced by H_2_O_2_ treatment was abolished by Dnmt3a overexpression. Similarly, a recent study by Li et al. [24] reported that H_2_O_2_ treatment inhibited DNA methylation by decreasing DNMT-1 levels in CD4^+^ T cells from patients with active lupus. Taken together, these findings of the present study suggested that decreased methylation levels of ALP and RUNX2 may be mediated by Dnmt3a downregulation in the context of OS.

There is growing evidence for an important role of DNA methylation in the regulation of osteogenic differentiation [41]. A recent study by Ha et al. [42] reported that nanohydroxyapatite modulated osteoblastic differentiation by the alteration of DNA methylation of the ALP promoter region. Zhang et al. [19] found that the hypomethylation of CpG islands in the promoter region of several osteogenic genes, including RUNX2, was associated with the osteogenic differentiation of mouse MSCs. Moreover, previous studies suggest that 5-AZA can improve osteogenic differentiation through reducing DNA methylation of osteogenesis-related genes [38, 39]. Consistent with these observations, our results demonstrated that 5-AZA, a Dnmt inhibitor, enhanced osteogenic differentiation through decreased DNA methylation levels of ALP and RUNX2 concomitant with increased expression of ALP and RUNX2 in the context of OS. Nishikawa et al. [20] recently found that S-adenosylmethionine-mediated DNA methylation by Dnmt3a regulated osteoclastogenesis via epigenetic repression of antiosteoclastogenic genes. Moreover, a more recent study by Liu et al. [21] suggests that Dnmt3a is involved in bone resorption and formation in myeloma. These studies suggest that Dnmt3a plays a key role in the regulation of bone remodeling. Therefore, 5-AZA-induced hypomethylation of ALP and RUNX2 contributes to osteogenic differentiation, as shown by the increased expression of ALP and RUNX2, which is likely to synergize with Dnmt3a downregulation induced by OS.

Excessive ROS suppress cell differentiation of endothelial progenitor cells, as shown by our prior study [43]; however, low levels of ROS exhibit a physiological intracellular signalling role [44], leading to survival, proliferation, and differentiation [3, 45, 46]. The effect of OS on osteogenic differentiation is extremely complicated, depending on ROS concentration and cell type. Intensive OS impairs osteogenesis of porous titanium implants [47], even resulting in implant failure; however, appropriate OS stimulates osteogenic differentiation [48]. Administration of antioxidants, especially in the early stage after implantation, is beneficial in the process of bone formation. Sandukji et al. [49] found that the ALP activity and osteocalcin level were markedly increased, whereas thiobarbituric acid reactive substances were significantly lower in the plasma of patients who received antioxidants for 2 weeks compared with those who did not receive antioxidants. Therefore, they concluded that the administration of antioxidants, including vitamins A, C, E, and selenium, could accelerate bone formation after the surgery of fracture fixation, and antioxidants should be considered in postoperative therapeutic protocols. The principles of bone tissue engineering involving scaffolds, drugs, and osteoprogenitor cells are widely used for the concepts of complex bone regeneration. In the future, we intend to investigate the effect of the biomimetic 3D scaffolds combined with antioxidants or 5-AZA on osteogenesis after implantation using an animal experiment.

## 5. Conclusion

The present study demonstrated that OS-induced DNA methylation changes mediated by Dnmt3a regulated osteogenic differentiation using a novel 3D cell culture model of hMSCs seeded in the scaffolds mimicking the microenvironment after implantation. Dramatic OS is harmful, whereas appropriate OS contributes to osteogenesis. Furthermore, we confirmed that 5-AZA can enhance osteogenic differentiation of hMSCs via the hypomethylation of ALP and RUNX2 under OS. In the field of bone tissue engineering, the 3D porous mineralized collagen scaffolds modified with antioxidants and 5-AZA may serve as a novel approach to improve osteogenesis after implantation.

## Figures and Tables

**Table 1:** Nucleotide sequences of the RT-PCR primers used.

Gene	Forward primer	Reverse primer
ALP	5′-ACCATTCCCACGTCTTCACATTT-3′	5′-AGACATTCTCTCGTTCACCGCC-3′
RUNX2	5′-ACTTCCTGTGCTCGGTGCT-3′	5′-GACGGTTATGGTCAAGGTGAA-3′
BSP	5′-AATGAAAACGAAGAAAGCGAAG-3′	5′-ATCATAGCCATCGTAGCCTTGT-3′
OPN	5′-GTCTCAGGCCAGTTGCAGCC-3′	5′-GCCATGTGGCCACAGCATCTG-3′
Dnmt1	5′-GGAGGGCTACCTGGCTAAAG-3′	5′-TACGGGCTTCACTTCTTGCT-3′
Dnmt3a	5′-GGGACGGAAAGAGAGAGACA-3′	5′-CCTGCTGCTAACTGGGTTCT-3′
Dnmt3b	5′-GAGCGGGCGGCAACG-3′	5′-CCCTTCATGCTTTCCTGCCG-3′
GAPDH	5′-TCGACAGTCAGCCGCATCTTCTTT-3′	5′-GCCCAATACGACCAAATCCGTTGA-3′

ALP: alkaline phosphatase; RUNX2: runt-related transcription factor 2; BSP: bone sialoprotein; Dnmt1: DNA methyltransferase 1; Dnmt3a: DNA methyltransferase 3a; Dnmt3b: DNA methyltransferase 3b; OPN: osteopontin; GAPDH: glyceraldehyde 3-phosphate dehydrogenase.

**Table 2:** The ratios of Dnmt expression at different time points in the ALIF model of pigs after implantation of COLLOSS® E and AUTOGRAFT.

	Time point	COLLOSS® E	AUTOGRAFT
Dnmt3a	2 w/4 w	0.376	0.757
	2 w/8 w	0.334	0.681
Dnmt3b	2 w/4 w	1.56	0.671
	2 w/8 w	0.850	1.25
Dnmt1	2 w/4 w	0.975	1.10
	2 w/8 w	1.51	1.08

COLLOSS® E: equine bone protein extracts; AUTOGRAFT: autograft bone; ALIF: anterior lumbar interbody fusion.

## Data Availability

The data used to support the findings of this study are available from the corresponding author upon request.
